# Supplementary material for: Successful Treatment of an Acinar Pancreatic Carcinoma in an Inland Bearded Dragon (Pogona vitticeps): A Case Report
Source: Animals (Basel). 2024 Jul 4;14(13):1976. doi: 10.3390/ani14131976 (PMC11240475; doi:10.3390/ani14131976)
Supplement: Supplementary file 1 [file animals-14-01976-s001.zip › animals-2952889-supplementary.pdf]

PRISMA 2020 flow diagram for new systematic reviews which included searches of databases and registers only

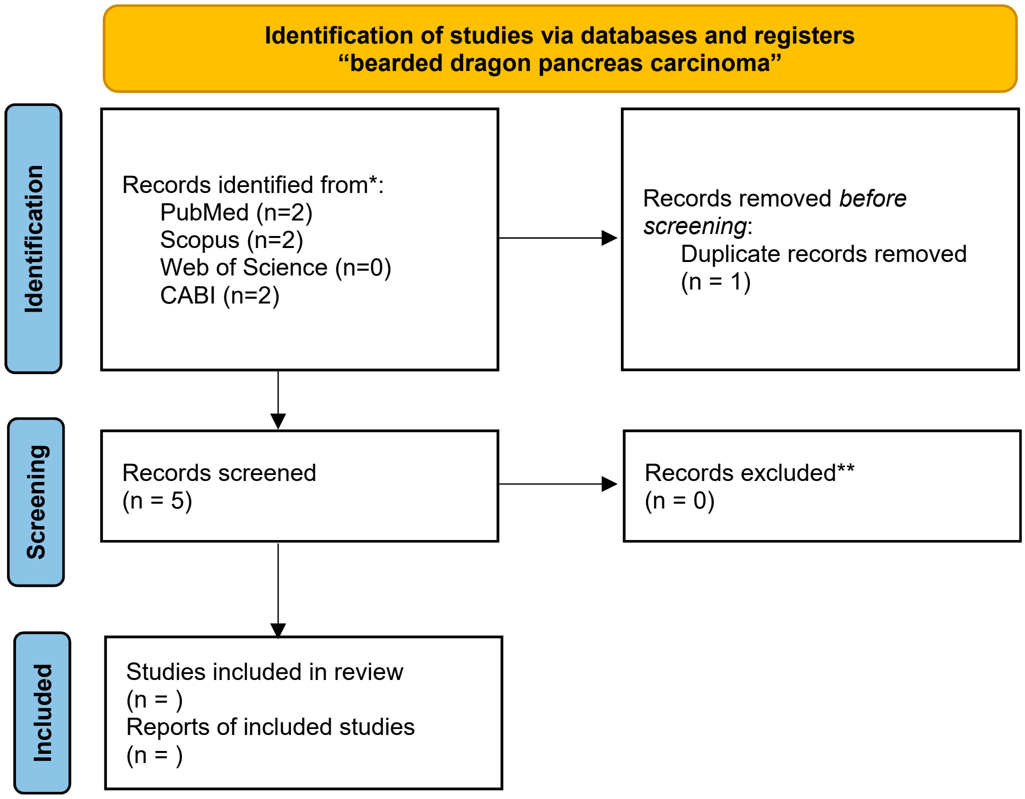

*Consider, if feasible to do so, reporting the number of records identified from each database or register searched (rather than the total number across all databases/registers).

**If automation tools were used, indicate how many records were excluded by a human and how many were excluded by automation tools.

From: Page MJ, McKenzie JE, Bossuyt PM, Boutron I, Hoffmann TC, Mulrow CD, et al. The PRISMA 2020 statement: an updated guideline for reporting systematic reviews. BMJ 2021;372:n71. doi: 10.1136/bmj.n71
